# Herbaceous Forage and Selection Patterns by Ungulates across Varying Herbivore Assemblages in a South African Savanna

**DOI:** 10.1371/journal.pone.0082831

**Published:** 2013-12-16

**Authors:** Anna Christina Treydte, Sabine Baumgartner, Ignas M. A. Heitkönig, Catharina C. Grant, Wayne M. Getz

**Affiliations:** 1 Agroecology in the Tropics and Subtropics, University of Hohenheim, Stuttgart, Germany; 2 Resource Ecology Group, Wageningen University, Wageningen, the Netherlands; 3 Systems Ecology, Scientific Services Kruger National Park, Skukuza, South Africa; 4 Environmental Science, Policy and Management, University of California, Berkeley, Berkeley, California, United States of America; 5 School of Mathematical Sciences, University of KwaZulu-Natal, Durban, South Africa; Institut Pluridisciplinaire Hubert Curien, France

## Abstract

Herbivores generally have strong structural and compositional effects on vegetation, which in turn determines the plant forage species available. We investigated how selected large mammalian herbivore assemblages use and alter herbaceous vegetation structure and composition in a southern African savanna in and adjacent to the Kruger National Park, South Africa. We compared mixed and mono-specific herbivore assemblages of varying density and investigated similarities in vegetation patterns under wildlife and livestock herbivory. Grass species composition differed significantly, standing biomass and grass height were almost twice as high at sites of low density compared to high density mixed wildlife species. Selection of various grass species by herbivores was positively correlated with greenness, nutrient content and palatability. Nutrient-rich *Urochloa mosambicensis* Hack. and *Panicum maximum* Jacq. grasses were preferred forage species, which significantly differed in abundance across sites of varying grazing pressure. Green grasses growing beneath trees were grazed more frequently than dry grasses growing in the open. Our results indicate that grazing herbivores appear to base their grass species preferences on nutrient content cues and that a characteristic grass species abundance and herb layer structure can be matched with mammalian herbivory types.

## Introduction

In African savannas, large mammalian herbivores have evolved to cope with generally low, seasonally varying quality of patchily distributed grasses and woody plants [1]. Certain minimum requirements such as nutrient thresholds for maintenance must be met [2] and various models have been developed on herbivore nutrient intake and food selection [3], [4]. Mammalian herbivores show specific forage requirements and preferences [5]; for instance, zebra *Equus burchelli* and African buffalo *Syncherus caffer* frequently graze on tall grasses [6], often of low nutrient content [7] while wildebeest *Connochaetes taurinus* prefer short and nutrient rich grasses [8]. Mixed feeders such as impala *Aepyceros melampus* feed on high quality grasses of different heights while switching to browse as grass nutrient quality declines [9] whereas pure browsers such as kudu *Tragelaphus strepciceros* or giraffe *Giraffa camelopardis* select mainly woody plant forage high in nutrients but also tannins [10]. It remains controversial, however, whether nutrient content, greenness or height are the most important cues used by various grazer species to identify the palatability of grasses [11], [12]. We, thus, claim that

H1: *Grass species that exhibit high nutrient content, palatability and greenness will be selected for by herbivores more strongly than others.*

Vegetation structure and composition is affected by herbivore density and foraging patterns [13] as well as preferences for particular plant species [14], [15]. These effects can be direct through differential removal and cropping of plant species or indirect by altering woody and herbaceous plant competition. For example, cattle at high densities can severely reduce grass cover while at moderate densities they stimulate grass productivity [16], [17]. Intensively grazing cattle are further known to promote the growth of “increaser species” (*sensu* [18]), i.e., mainly stoloniferous and grazing-tolerant species. Hence, we expect that

H2: *Only few and grazing resistant species will dominate areas of mono-specific grazing, particularly at high grazer densities, in contrast to a higher grass species diversity expected in areas of mixed herbivore assemblages.*

Grass height is influenced by the individual grass species properties but is also likely to differ depending on the present grazer species assemblage. Cattle alone, particularly at high densities, graze the grass layer short, i.e., close to ground level [19] without strong grass selection [20]. Wild herbivores, in contrast, freely move on grazing grounds and their forage intake depends mainly on their mouth width and body weight [13], which shapes the vegetation accordingly. Research has investigated how various densities or full exclosures of one herbivore type shape the vegetation [21], [22] but the influence of selectively enclosed mono-specific and multi-species herbivore communities shaping herbaceous layer vegetation has received less attention. A more diversified grazing pressure across varying plant species might lead to a higher species and structural heterogeneity within the herbaceous layer, in which grazer and browser impacts on the vegetation are more evenly distributed [23], [13]. Further, browsing herbivores influencing woody vegetation indirectly affect grazer distributions because herbaceous layer characteristics depend on micro-climatic and soil-nutrient benefits provided by tree canopies [24]. Assemblages of mixed wild ungulate species can then use that heterogeneity in the vegetation at various spatial scales within the landscape [25]. While studies found that cattle grazing favored short-growing, grazing-resistant grass forms [26], little comparative studies exist for the impact of mono-specific wild grazers and a mixture of grazers and browsers on the vegetation. We hypothesize that

H3: *Grass standing biomass, height, and cover as well as their leaf nutrient contents will be higher at mixed herbivore assemblage sites compared to areas of mono-specific grazing.*

Hence, in areas of higher grass biomass, grass nutrients might also be enhanced despite reported potential nutrient dilution effects [27]. Therefore, we further expect that

H4: *Grass leaf nitrogen and phosphorus contents will be higher in areas of higher grass standing biomass, i.e., in areas of lower grazing pressure.*

Similar patterns might arise for the wild and domestic herbivore counterparts when comparing the effect of mono-specific and multi-species assemblages on plants. Hence, comprehensive studies comparing the effects of wild grazers only with mixed browser-grazer assemblages of similar densities are needed to understand whether livestock management can learn from wildlife foraging ecology. As large scale, controlled studies are logistically difficult insights can also be obtained from smaller scale studies. In our case, these take advantage of already grazed areas, matched in their ecological characteristics, but entailing different grazer assemblage histories. Here, we explore whether preferred grass species are similar across herbivore assemblages (wild, domestic, mono-specific or mixed). We investigate the effect of different herbivore communities and densities on the grass species composition and structure in and around Kruger National Park, South Africa.

## Methods

Our field studies were carried out with permission of South African National Parks (SANPARKS, project ID: TREAC 881) and with approval of the local chiefs, Mnisi Tribal Authority, Bushbuckridge Municipality. The study did not involve endangered or protected species and no animal species were sacrificed or impeded in any way in their normal behaviour. Our study focused on vegetation only and we did not conduct direct behavioural observation or animal experiments but based our data on indirect observation techniques only (see below).

### Study area

Our study was carried out in and around the Satara area (24°22′S, 31°46′E) in Kruger National Park (KNP), South Africa, during April-June 2011, shortly after the rainy season. Here, annual average rainfall is 550 mm [28] (Scientific Services, meteorological records), with a bimodal rainfall pattern. Dominant tree species are *Acacia* spp., *Sclerocarya birrea*, and *Combretum* spp. while grasses are predominantly composed of *Bothriochloa* spp, *Themeda triandra*, *Urochloa mosambicensis*, *Digitaria eriantha*, and *Aristida* spp.[29], [24].

To compare different grazer and browser herbivore assemblages and to identify whether similar patterns can be detected for wildlife and livestock herbivory we selected five sample sites (Table 1: mono-specific wildlife  =  MonoW, only dominated by African buffalo; mixed species wildlife of low density  =  MixWLow; mixed species wildlife of high density  =  MixWHigh; mono-specific livestock  =  MonoL, viz., cattle grazing area; mixed livestock area  =  MixL).

**Table 1:** Study sites and their location, herbivore assemblage type, feeding guilds and densities (in tropical livestock unit TLU ha^−1^).

Designation	Location	Herbivore type	Feeding guild	Herbivore density*	# of transects	# of plots
MonoW	KNP	Wildlife	Grazer	0.1	17	24
MixWLow	KNP	Wildlife	Browser & Grazer	0.1	20	24
MixWHigh	KNP	Wildlife	Browser & Grazer	0.9	21	24
MonoL	Mnisi	Livestock	Grazer	0.9	14	24
MixL	Mnisi	Livestock	Browser & Grazer	0.9	14	24

=  mono-specific wildlife site, MixWLow and MixWHigh  =  mixed-species wildlife sites of low and high herbivore densities, respectively; MonoL and MixL  =  mono-specific and mixed-species livestock sites of high density, respectively. The total number of transects and plots for grazing impact recording and herbaceous layer assessment, respectively, is given. KNP  =  Kruger National Park, Mnisi  =  communal grazing land outside of KNP. MonoW

*Herbivore density measured in TLU ha^−1^

Three wildlife study sites were located inside KNP with largely uniform soil conditions, topography and fire frequencies. The MonoW site, a 870 ha enclosure that had been fenced in the 1990s to breed tuberculosis-free buffalo, hosted a current population of about 96 African buffalo at an average density of 0.11 TLU ha^−1^ [24]. Adjacent to the enclosure mixed grazer and browser assemblages were dominated by zebra, wildebeest, giraffe, and impala [29], [24]. Within a 5 km radius of the enclosure, we selected one area of high herbivore density (up to 0.9 TLU ha^−1^ close to the Satara water hole [30]) and one of low density (0.1 TLU ha^−1^ directly adjacent to the enclosure; [24]), with densities being affected by distance to water on a seasonal basis [31], [32].

The two livestock study sites were outside of KNP, about 35 km away from the buffalo enclosure, in the Mnisi district, Mpumalanga, South Africa. Here, livestock density of the communal grazing land is approximately 0.88 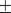 0.09 TLU ha^−1^, close to local ecological carrying capacity and exceeding recommended stocking rates by about 400% [33], [34]. In contrast, browser stocking rate exploits only 50% of the potential browsing capacity [35]. The selected sites were less than 1 km apart and similar in slope, aspect, and rainfall regime to the KNP sites. One area was dominated by cattle grazing only (cattle breed: *Bos taurinus*; [36]) and an adjacent site was grazed and browsed regularly by cattle, few sheep and domestic goats (Veterinary Services, unpublished). Wildlife herbivore sites were dominated by basalt with haplic luvisols [37] while livestock areas were located on granites with sandy lithosols [34].

Along plots and transects we assessed the herbaceous layer structure and composition, respectively. (1) Plots: We determined 24 sample plots of 1 m×1 m along four W-E lines within each study site, which were 50 m apart and parallel to each other. As vegetation structure and forage quality might differ between tree-influenced areas (“sub-canopy”) and areas outside the influence trees (“outside canopy”), 12 sample plots each were located in the sub-canopy and in the outside canopy area, respectively. (2) Transects: We selected 20 m long transects to assess grass species, height and signs of grazing , radiating away from vegetation plots along a N-S line, which was not always achievable due to bush thickets, resulting in different numbers of transects (14–21) across sites (Table 1).

### Data collection

Every m along each transect, we identified the tufts of grass species touching the transect line, measured tuft height, visually assessed their greenness (on a level of 0 =  dry, i.e., entire plant parts brown; 1 =  green, i.e., roughly 50% of the plant parts green; 2 =  very green, i.e., most plant parts freshly green), and noted their sub-canopy or outside canopy location. In addition to these parameters we recorded whether or not the grass tufts had been grazed (>5 stems or leaves of the grass tuft bitten off at the same height level and cuts being planar; 1 =  grazed, 0 =  not grazed).

Within each vegetation plot, we estimated overall ground cover visually and assessed standing biomass using a Disc Pasture Meter, calibrated for KNP [38]. Within each 1 m×1 m vegetation plot, we estimated grass species abundance and forb cover by eye to the nearest 5% [39]. We identified species with the help of local experts and van Oudtshoorn (2004) [40] and calculated the Shannon diversity index. At each plot, we sampled the most dominant grass species summing up to 80% of cover from at least three separate individual grass tufts; we separated samples into stem and leaf, dried the latter to constant weight and analysed them at the Soil Science Institute, University of Hohenheim, for Nitrogen (N) with an Elementar VarioMacro Analyzer (catalytic oxidation with subsequent N analyses via thermal conductivity detector) and Phosphorus (P) after microwave digestion and photometric analysis using a Varian UV-Visible Spectrophotometer (DIN EN 1189).

We gave each grass species a grazing value score according to [40] who determined grazing value for cattle as the grass species' ability to produce leaf material, its digestibility, nutritional value and growth vigour, i.e., capacity of regrowth after grazing. We classified this species-specific grazing value into three levels: 0 =  unpalatable, 1 =  moderately palatable, 2 =  highly palatable and used it in further analyses (Table 2).

**Table 2:** Comparisons conducted and their statistical values addressing the various questions (see Table 1 for abbreviations).

Question	Contrast or Test	Statistic	Significance		
*Grass height in non-grazed sites*					
	MixL vs. MonoL	*F*_1,279_ † = 65.3		*P*<0.001	
	MonoW vs. (MixWLow and MixWHigh)	*F*_2,360_ † = 39.4		*P*<0.001	
*Grass height in grazed sites*					
	MixL vs. MonoL	*F*_1,241_ † = 87.9		*P*<0.001	
	MonoW vs. (MixWLow and MixWHigh)	*F*_2,264_ † = 59.5		*P*<0.001	
*Grass leaf nutrients*					
	nitrogen content vs. biomass	*R* ^2^ ‡ = 0.40		*P* = 0.05	
	phosphorus content vs. biomass	*R* ^2^ ‡ = 0.74		*P*<0.001	
	nitrogen content in MixL vs. MonoL	*F*_3,25_ ** = 9.23		*P*<0.001	
	nitrogen content in MonoW vs. (MixWLow and MixWHigh)	*F*_5,34_ ** = 3.93		*P* = 0.006	
*Grazer preference*					
	nitrogen content vs. grass grazed	*R* ^2^ ‡ = 0.43 (df = 14)		*P* = 0.008	
	palatability vs. grass grazed	*χ* ^2^ * = 7.9 (n = 17)		*P* = 0.019	

See also figures 4 and 5 for trends and values.

† One-way ANOVA with Tukey's-HSD post-hoc test

‡ Simple linear regression

*Kruskal-Wallis test

**nested ANOVA (canopy nested within site)

### Data analyses

We derived total species richness, Shannon-Wiener diversity index, evenness, and species distribution from data on species presence and abundance. Analysis of Similarity (ANOSIM; Past version 2.10) tested for differences in herbaceous layer species composition and Bray-Curtis distances for comparisons between groups [41]. Non-parametric multi-dimensional scaling analyses illustrated similarity and dissimilarity among sample sites and identified those species that exerted strongest influence on dissimilarities between sites [42]. As herbivore densities differed across wildlife and livestock-dominated areas, we treated these areas separately in our ANOVA analyses. Nested ANOVA tested the influence of the fixed factors “canopy” nested within “site” and their interaction on herbaceous vegetation. One-way ANOVA and Tukey-HSD post-hoc tests compared the average grazed and ungrazed height of grass tufts across transects at the different sites [43]. On aggregated site values, we applied linear regressions and Kruskal-Wallis tests to identify whether grass nutrient contents were related to estimates of palatability and whether grazed tuft numbers were correlated with grass nutrients or structure. We tested grass tuft locations along transects (sub- or outside tree canopy) using a *χ^2^* test, calculated as follows: out of 1722 location points along the transects, 485 locations ( = 28%) were found beneath trees. Hence, for each grass species, we expected 28% of individual tufts to grow beneath trees. Thus, we multiplied the total number of tufts found per species by 0.28 as expected value and compared with the observed proportion of grass tufts found beneath trees. Further, on 36% of all location points along transects, the grass tufts were grazed; hence, we multiplied the total individual tuft number per species by 0.36 as expected value and compared with observed values on grazed grass tufts using a *χ^2^* test. We transformed data that did not fit normal distribution assumptions accordingly [43]. Statistical analyses were conducted using SPSS (PASW Statistics 18).

## Results

### H1: Grazers prefer grasses of high nutrients, palatability and greenness

**Figure 1:**
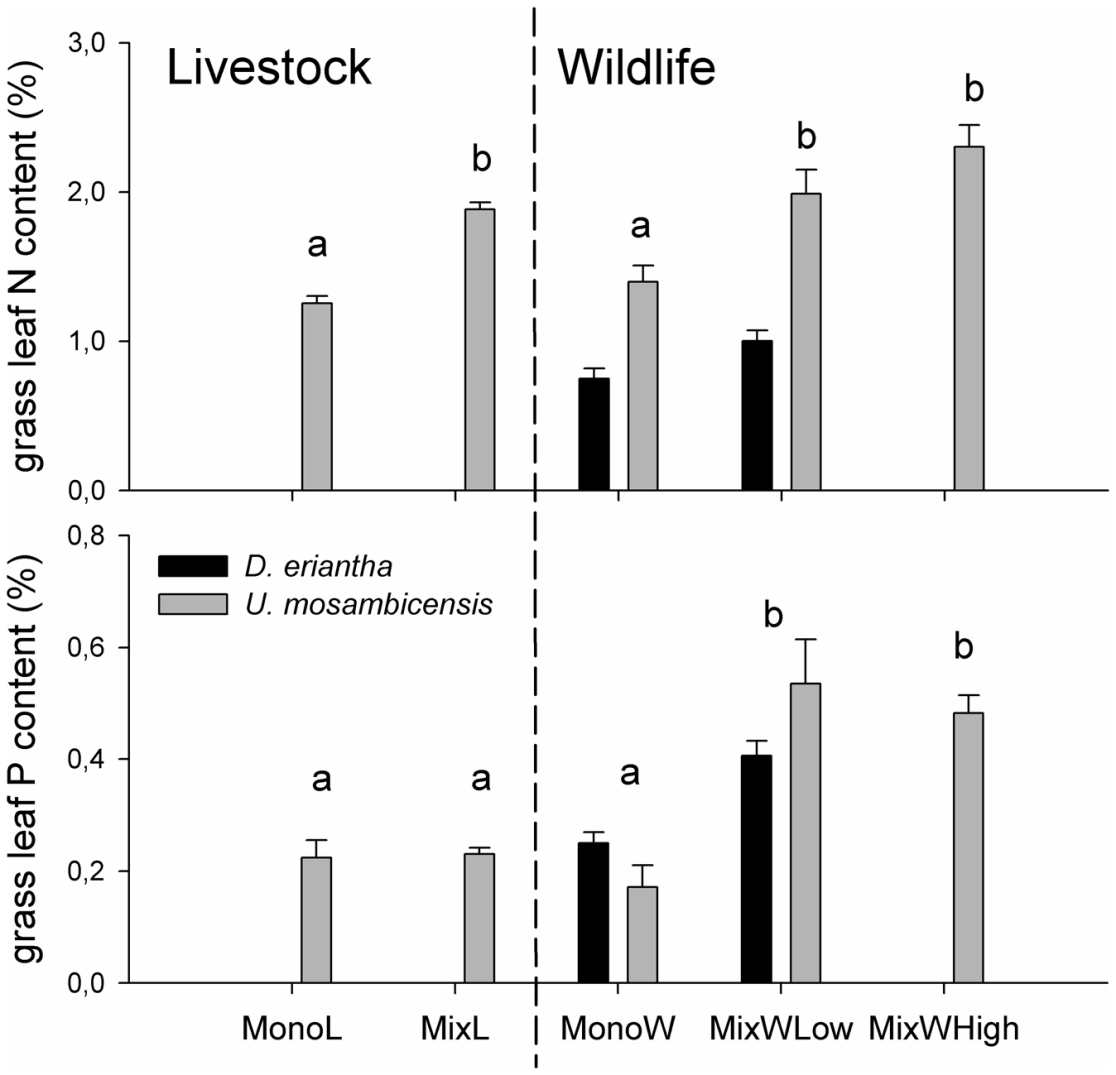
Average (±SE) grass leaf nitrogen (N) and phosphorus (P) content of *Urochloa mosambicensis* and *Digitaria eriantha* across study sites (for abbreviations see Table 1). Different letters denote significant differences of the mean (HSD-Tukey).

Grass leaf nutrient contents of *D. eriantha* and *U. mosambicensis* differed significantly across study sites as did overall nitrogen contents for KNP and Mnisi areas (Table 2). Grass leaf N content of *U. mosambicensis* was by about 1/3 significantly higher in mixed than in mono-specific herbivore assemblage sites (*F_4,66_* = 20.9, *P*<0.001) while patterns were less strong for grass leaf P contents (*F_4,65_* = 16.1, *P*<0.001; Figure 1).

**Figure 2:**
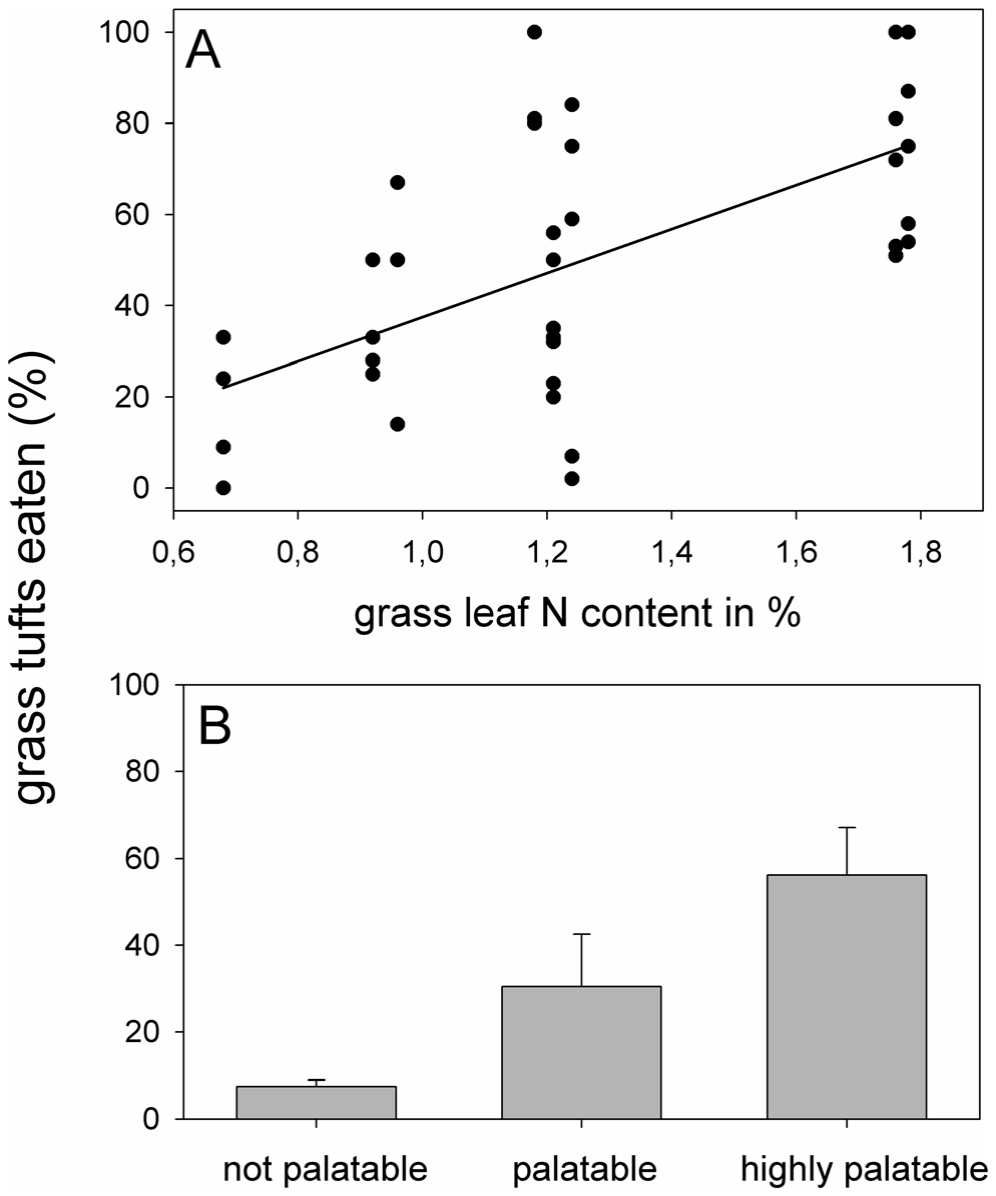
Percent of grazed grass tufts versus (A) grass leaf N content in % dry matter and versus (B) ranked palatability.

**Figure 3:**
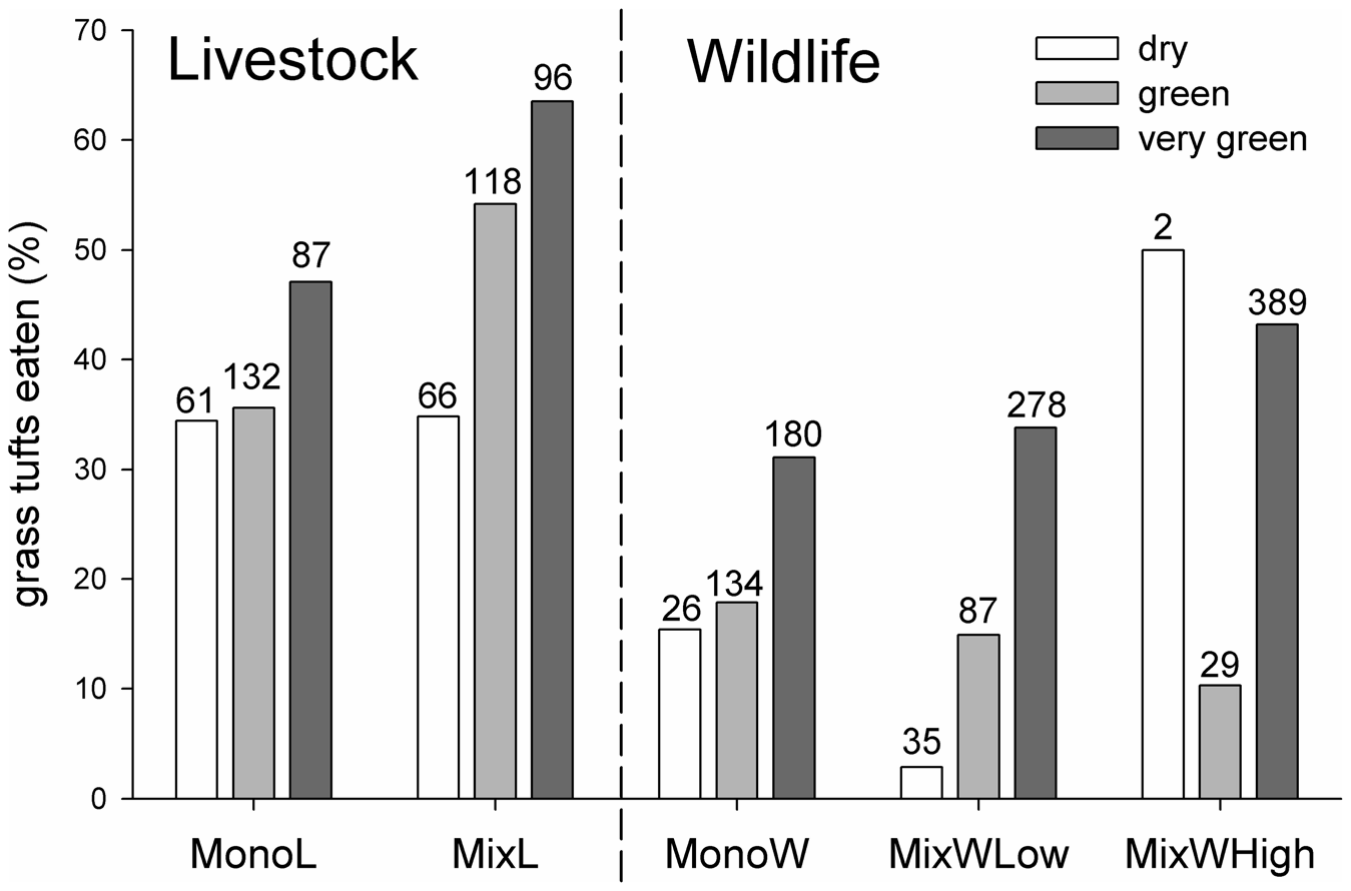
The average percentage of grazed grass tufts according to their absolute greenness values across the five study site types defined in Table 1. Numbers above bars indicate sample size.

Generally, recorded grass leaf N contents were positively related to the number of times grass species were grazed (*y* = 48.4*x* − 10.9; Table 2, Figure 2A). Grasses in the higher palatability classes tended to be grazed more frequently than grasses of lower palatability (Table 2, Figure 2B). Further, mammalian herbivores were feeding more frequently on green rather than dry grasses, based on absolute values (*F_1,25_* = 8.25, *P*<0.01), and wildlife grazed proportionally less on dry grasses compared to livestock, the latter including at least 30% of dry grasses in their diet (Figure 3).

*Urochloa mosambicensis* was grazed more than 50% of the time, more frequently than expected from the percentage occurrence, and more strongly so in wildlife areas (Table 3). The same was true for *Panicum maximum*; *Bothriochloa* spp. were strongly grazed at livestock sites (Table 3). Despite its relatively high abundance, *Themeda triandra* was grazed less often in wildlife than in livestock areas (Table 3). *Eragrostis* spp. were grazed more frequently than expected while *Aristida* spp. and *D. eriantha* were grazed only 30–50% of the time, hence, not preferred by either livestock or wildlife. *Panicum coloratum* and *Heteropogon contortus* were neither preferred nor strongly rejected (Table 3).

**Table 3:** Average grass leaf nitrogen (N), phosphorus (P), increaser (I) or decreaser (D) species and palatability (Palat.code: 0 =  not palatable, 1 =  moderately palatable, 2 =  highly palatable) with respect to veld condition [42].

				Palat.	MonoL		MixL			MonoW		MixWLow		MixWHigh	
Grass species	I/D	% N	% P	code	n	% g	n	% g		n	% g	n	% g	n	% g
*Aristida* spec.	I	0.68		0	45	33	59	24		13	0	10	0	36	9
*Bothriocloa* spec.	I	1.24		1	8	75	51	84		122	7	54	2	188	59
*Cenchrus ciliaris*	D	1.18		2						3	100	15	80	27	81
*Digitaria eriantha*	D	0.92	0.19	2	9	33				28	25	110	28	2	50
*Eragrostis* spec.	I			1	34	78	16	83		1	100	2	0	17	100
*Heteropogon contortus*	I	0.96		1	6	50	18	67		1	0	7	14	2	50
*Melinis repens*	I			0	10	20	3	0							
*Panicum coloratum*	D	1.21		2	1	100	1	0		35	20	38	32	12	33
*Panicum maximum*	D	1.78		2	4	75	2	100		59	54	19	58	23	87
*Pogonarthia squarrosa*	I			0	13	15	1	0							
*Setaria verticillata*	D			1	8	25	13	92							
*Themeda triandra*	D	1.21		2	16	56	2	50		52	35	110	23	1	0
*Urochloa mosambicensis*	I	1.76	0.23	2	105	51	101	53		4	100	18	72	72	81

Individual grass tuft abundance (n) and percentage of the individual tufts grazed (% g) along transects are given across the various site types (see Table 1 for abbreviations). Forbs and grasses of overall <5% relative abundance are not included.

### H2: Low grass species diversity at mono-specific grazing sites

In the wildlife dominated area, MixWLow showed highest species richness for both sub- and outside canopy areas (Table 4) and highest diversity; at MonoW, diversity was more than 40% lower, particularly for grass assemblages growing outside tree canopies (Table 4A). At livestock sites, grass species richness did not differ significantly while diversity tended to be slightly higher in MixL compared to MonoL (Table 4B). Grass species composition differed significantly among wildlife sites (ANOSIM: *R* = 0.39; *P*<0.001) but less strongly amongst livestock dominated sites (*R* = 0.17; *P*<0.001).

**Table 4:** Mean values of herbaceous layer species richness, diversity, biomass and cover at wildlife (A) and livestock (B) sites (see Table 1 for abbreviations; in addition, “sub” represent sites influenced by tree canopies while “out” are sites outside the sphere of influence of tree canopies).

A.	Canopy	MonoW	MixWLow	MixWHigh	Site effect	Canopy effect
Species richness	out*	3.5^a^±1.6	5.0^b^±1.2	4.4^ab^±1.2	*F*_2,33_ = 4.4; *P* = 0.020	
	sub*	4.8^ab^±1.5	5.3^b^±1.2	4.0^a^±0.7	*F*_2,33_ = 3.7; *P* = 0.037	
Shannon-Wiener diversity	out*	0.80^a^±0.49	1.33^b^±0.21	1.12^ab^±0.27	*F*_2,33_ = 6.9; *P* = 0.003	
	sub*	1.27^a^±0.35	1.34^a^±0.28	1.10^a^±0.27	*F*_2,33_ = 2.0; *P* = 0.155	
Standing biomass [kg/ha]	out‡	3215±842	4251±706	2434±1989	*F*_2,33_ = 13.0	*F*_1,33_ = 0.2
	sub‡	2494±1087	4147±695	2929±851	*P*<0.001	*P* = 0.678
Cover [%]	out*	59^a^±15	82^b^±7	52^a^±27	*F*_2,32_ = 7.5; *P* = 0.002	
	sub*	48^a^±22	78^b^±9	78^b^±19	*F*_2,33_ = 11.3; *P*<0.001	

B.		MonoL	MixL			
Species richness	out‡	5.3±1.4	5.4±0.9		*F*_1,46_ = 0.1;	*F*_1,46_ = 0.6;
	sub‡	5.7±1.3	5.6±1.2		*P* = 0.856	*P* = 0.464
Shannon- Wiener diversity	out‡	1.22±0.4	1.32±0.22		*F_1,46_* = 4.0;	*F*_1,46_ = 1.4;
	sub‡	1.25±0.3	1.49±0.21		*P = *0.051	*P* = 0.250
Standing biomass [kg/ha]	out‡	1334±534	526±420		*F*_1,23_ = 19.2	*F*_1,23_ = 29.1
	sub‡	2179±871	1156±562		*P*<0.001	*P*<0.001
Cover [%]	out‡	69±20	64±23		*F*_1,23_ = 0.3	*F*_1,23_ = 7.3
	sub‡	80±17	78±16		*P* = 0.570	*P* = 0.013

*F* and *P* statistics of one-way ANOVA are given for separate and full (for significant interactions) models on herbivore treatments (Site effect), influence of canopy (Canopy effect) and their interaction. Different letters indicate Tukey-HSD significant differences at *P* = 0.05.

*significant interaction between site and canopy; *F*- and *P*-value given for the full model

‡ separate one-way ANOVA for different canopy categories were conducted.

At wildlife sites, *B. radicans, U. mosambicensis, T. triandra* and *D. eriantha*, all of intermediate to high nutrient and grazing value, contributed with 67% most to the distinction of species community among all three sample sites (Table 3). In the livestock dominated area, *U. mosambicensis* and *B. insculpta* contributed most (32% and 24%, respectively) to the differences in species composition between sites, together with the nutrient-poor *Aristida congesta* (17%). Nutrient-rich and high grazing value species such as *P. maximum* and *T. triandra* accounted with <12% for the difference amongst livestock sites (Table 3). *Digitaria eriantha* and *T. tiandra* were abundant at MonoW and MixWLow sites but less so at livestock sites. *Aristida* spp. and *E. superba* mainly occurred in areas of high grazing pressure as did the nutrient rich and palatable *U. mosambicensis*, which was particularly abundant in livestock sites (Table 3). The palatable and nutrient-rich *P. maximum* occurred more frequently at mono-specific and wildlife-dominated sites (Table 3).

Tree presence influenced the grass species abundance significantly (*χ^2^* = 138, *P*<0.001); *P. maximum* was recorded more than twice as frequently (*P*<0.001) while *B. radicans* was found 20% less frequently (*P*<0.001) than expected beneath trees. *Urochloa mosambicensis*, *T. triandra* and *D. eriantha* abundance did not strongly depend on tree canopies whereas *Aristida* spp. (*P*<0.001) and *Eragrostis* spp. (*P* = 0.042) were more frequently than expected found outside of tree canopy influence.

### H3: High grass biomass, height and cover at mixed herbivore assemblage sites

Within wildlife dominated sites, both standing herbaceous biomass and total herbaceous cover were significantly higher at MixWLow (Table 4). At livestock dominated sites, MonoL biomass was significantly higher than MixL, while total herbaceous cover did not differ significantly (Table 4). At wildlife sites, herbaceous biomass and cover showed a trend to be lower beneath tree canopies whereas the opposite was the case at livestock sites (Table 2). Forb cover did not differ strongly across site or canopy (*F*<0.2, *P*>0.8, *n* = 66).

**Figure 4:**
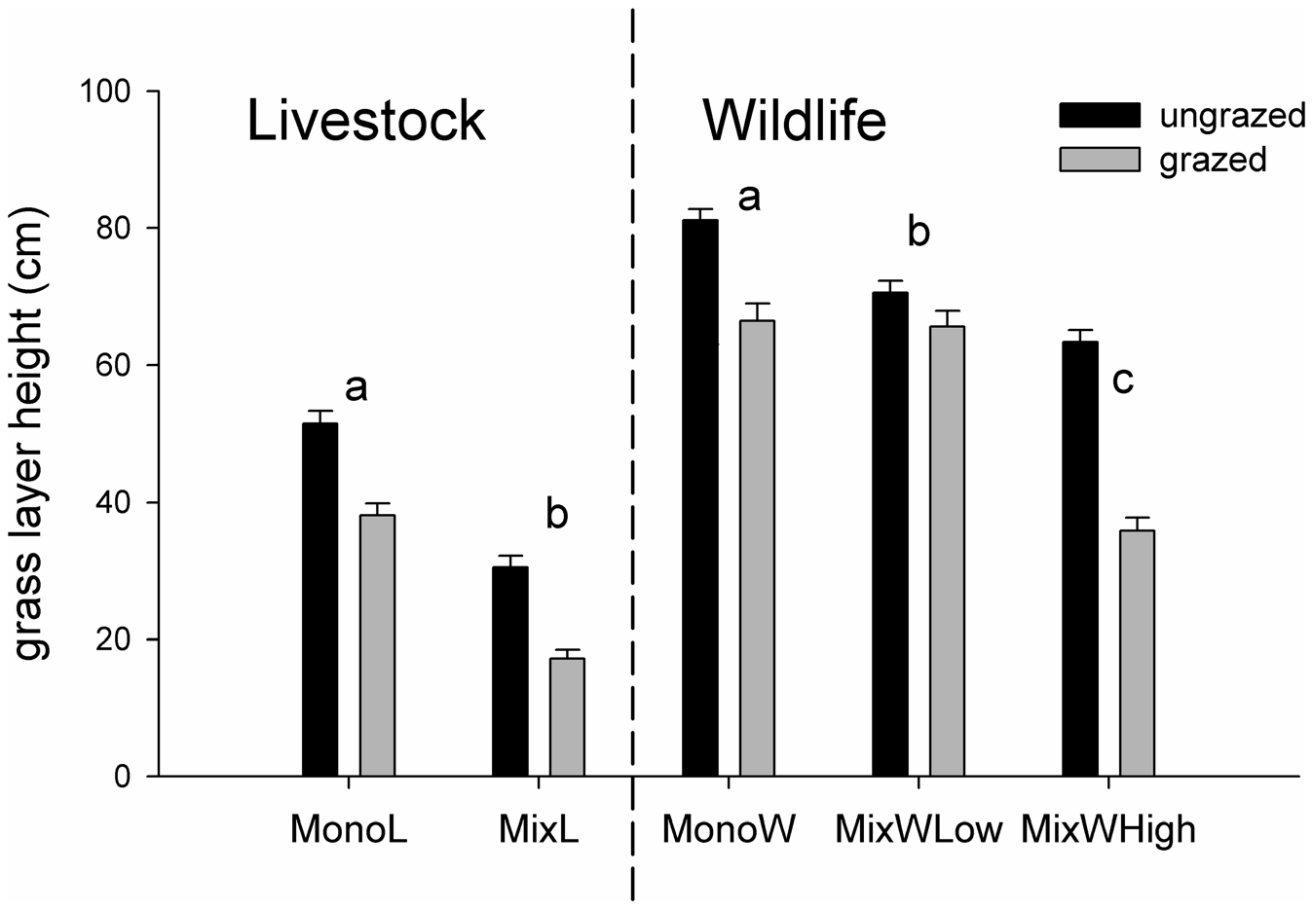
Average (±SE) grass height of ungrazed and grazed sites for different site types (see Table 1 for abbreviations). Different letters denote significant differences of the mean (HSD-Tukey).

Contrary to expectation, all individuals of non-grazed and grazed grasses were significantly taller in mono-specific versus mixed-species sites (Table 2, Figure 4). The MixWHigh grasses were reduced to almost half the size when grazed whereas grass heights were reduced by 10–20% through grazing at other sites (Figure 4). In wildlife dominated areas, non-grazed *Bothriochloa* spp. grew almost twice as tall (54 cm) at MixWHigh and MonoW compared to MixWLow (*F*_2,299_ = 45.4, *P*<0.001). Non-grazed *U. mosambicensis* grew almost twice as tall (68 cm) in MixWLow compared to MixWHigh (37 cm; *F*_1,17_ = 7.48, *P* = 0.014) and was grazed shortest (17 cm) at MixWHigh compared to MixWLow and MonoW (56 cm and 43 cm, respectively; *F*_2,72_ = 26.4, *P*<0.001). The same pattern was seen for *U. mosambicensis* at livestock areas as grazed grasses were with 18 cm about half as tall at MixL than at MonoL (*F*_1,106_ = 35.9, *P*<0.001). Grazed and non-grazed *T. triandra* heights did not differ across wildlife sites (*F*_1,41_ = 2.6, *P* = 0.117 and *F*_2,117_ = 0.3, *P* = 0.687, respectively) and abundance at livestock sites was too low for statistical analyses. Grazed *Panicum* spp. were with an average height of 59 cm more than 10 cm shorter at MixWHigh than at MonoW and MixWLow (*F*_2,83_ = 6.6, *P* = 0.002).

### H4: Grass leaf N and P increasing with grass biomass

**Figure 5:**
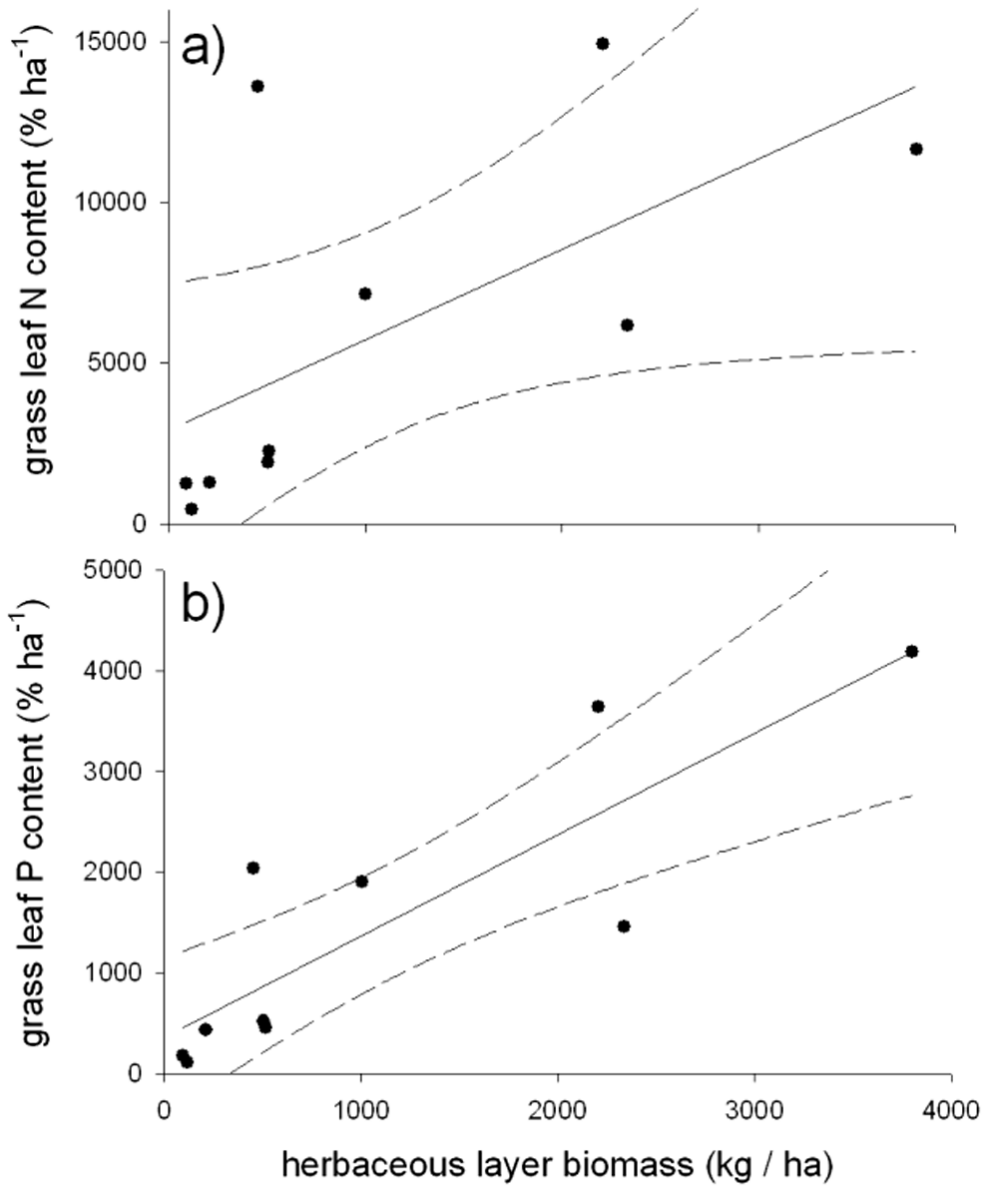
Regression line and 95% CI (solid and dashed lines, respectively) of grass leaf N (A) and P (B) contents against overall herbaceous layer standing biomass. Filled circles represent values for sub- and outside tree canopy herbaceous biomass averaged for each site.

Using herbaceous standing biomass estimates and grass species abundance beneath and outside tree canopies, taking into account the proportional canopy cover of trees at each site (see also [44]), we found that overall grass leaf N and P contents were positively correlated with available herbaceous layer biomass across all sites (for N: *y* = 2.82*x* + 2887; for P: *y* = 1.01*x* + 360; Table 2), with P showing stronger trends than N (Figure 5). Further, grass standing biomass was strongly positive associated with grass layer height (*y* = 0.02*x* + 19; *R^2^* = 0.81, *P*<0.001). As soil differences might have been a confounding factor influencing vegetation we also analysed soil water availability and nutrient contents at all study sites; variations were high and water infiltration rate did not differ statistically across sites nor did soil nitrogen contents (*F_4,28_* = 21.4, *P* = 0.270). Wildlife and livestock multi-species study sites had slightly lower penetration depth ( =  higher compaction) than mono-specific sites (*F_2,33_* = 2.9; *P* = 0.101, *F_1,23_* = 8.2; *P* = 0.009, respectively). Livestock sites had lower P contents compared to wildlife sites but variations were high (*F_4,31_* = 57.8, *P*<0.001).

## Discussion

### H1: Grazers prefer grasses of high nutrients, palatability and greenness

In our study, all grazers, whether wild, domestic, or mixed with browsers, strongly selected for green, palatable grasses of high nutrient content. *Urochloa mosambicensis* was highly abundant and frequently grazed by both wildlife and livestock. Hence, as predicted, it can persist under intense utilization (see also [45]) and might even accrue higher nutrient contents when browsers are included in the herbivore system: we showed that *U. mosambicensis* expressed up to twice as high N and P contents in mixed compared to mono-specific herbivore sites. Further, both wild and domestic species in our study preferred the nutritious and green *P. maximum*, a species that can be found across various savanna systems in eastern and southern Africa, particularly in shady spots [46], [47]. However, *P. maximum* might not persist under heavy grazing, when grazing-resistant annuals and stoloniferous grasses have a higher survival advantage [48]. This trend we also observed as *P. maximum* abundance was low at livestock-dominated sites. Further, with a current decline in large trees [49], [50], beneath which *P. maximum* preferentially grows, African rangelands are in danger of losing an important forage grass species in the future. Both *Bothriochloa radicans* and *B. insculpta*, of low nutrient but high phenolic contents, were low preference grass species in our study, despite their locally high abundance and average greenness, and contrary to other studies [40], [51]. *Bothriochloa* spp. spreads quickly when openings are created under high grazing pressure [52], which might explain their locally high abundance in wildlife and livestock sites. The value of *T. triandra* for rangeland conditions appears to lie rather in its ubiquitous presence in highly grazed areas [53], [51] than in its nutrient value. However, *T. triandra* could well play a role as a key resource during the dry season when more nutritious grasses have been depleted.

### H2: Low grass species diversity at mono-specific grazing sites

In our study, mixed herbivore sites, particularly under moderate wild herbivore pressure, encompassed the highest grass species diversity, which was expected since an assemblage of various selectively grazing herbivores can utilize the grass sward differently [5] and, thus, promotes both species and structural heterogeneity. Moderate herbivory was found to increase plant biodiversity in African savannas [45] while for a short-grass steppe in Colorado highest grass species diversity was found in the full absence of grazing [54]. In South Africa, high rainfall, light grazing and frequent bush fires promote *T. triandra* abundance [51], [53], which, in our study, was about five times more abundant in wildlife-dominated areas, particularly under low herbivore density. In contrast, mono-specific intensive grazing can lead to stands of only few dominant grass species, especially in combination with fire, as was shown for Australian [55] and African savannas [13]. Our findings agree with [13] who suggests that intense livestock grazing promotes short, stoloniferous and grazing resistant lawn grasses, in our case represented by *U. mosambicensis*, while moderate grazing increases patch heterogeneity, including short and tall grass species. In the wildlife-dominated area, perennial tufted plants such as *B. radicans*, *T. triandra* and *D. eriantha* were about half as abundant at the MixWHigh site compared to the other two sites. A similar trend, i.e., replacement of palatable and seed-producing grasses by stoloniferous grasses has been observed particularly under high mono-specific cattle grazing pressure [52]. Our observed patterns on grass species composition and the parallel patterns found in wildlife and livestock areas fit observations of other livestock [51] and wild herbivore [56] studies. Direct comparisons, however, have to be done with great care as sites were about 35 km apart and other confounding factors such as human impact might have additionally led to differences in grass species composition. Thus, we conducted separate analyses for wildlife and livestock sites. Further, we only covered plant characteristics during the growing season and further research on the effects of season and soil properties are needed to shed additional light on the importance of these factors in determining plant species composition.

### H3: High grass biomass, height and cover at mixed herbivore assemblage sites

Grass height differed strongly across sites and was, against our expectations, lower in mixed than in mono-species herbivore assemblages. Generally, wildlife areas of low grazing pressure (MonoW and MixWLow) showed highest biomass, cover and grass height. While grasses are usually grazed down to almost ground level [57], the average grazing amount in our study was with about 10 and 20% grass removal rather low. However, at our mixed-herbivore wildlife and livestock sites of high grazing pressure grasses were grazed down to half of their original height. This highlights the small-scale landscape heterogeneity that might have been created by (a) a variety of different grazing species removing grasses at various heights and, (b) a higher structural diversity of grass species per se. In general, variances of grazed grass height were larger than for ungrazed grasses, indicating that animals did not forage uniformly across space but used certain patches more intensely than others. As our study was conducted during the growing season, i.e., when grasses are still abundant, the already low average height of grasses at livestock sites might be of concern due to the lack of tall grass patches that represent important dry season resorts for grazing herbivores [13]. Our findings might have further implications for management because areas of shorter grasses are less prone to fire and have cooler burns, generating feedbacks on grass species composition itself [58].

### H4: Grass leaf N and P increasing with grass biomass

In our study, sites of higher biomass, i.e., under low herbivore density but also including browsers in the system, showed higher overall nutrient contents of the herbaceous layer than sites of lower herbaceous biomass. This was particularly visible for phosphorus contents, an often limiting nutrient in nutrient-poor savanna systems [59]. The fact that wildlife tends to select patches of higher nutrient content has been well documented for African savannas [60]–[62]. While higher nutrient intake in a more heterogeneous environment will result in higher livestock production the current management of livestock in African savannas still tends to encourage uniform grass sward utilization. This will likely decrease livestock productivity as we showed that domestic herbivores also select for specific grass species. Hence, we suggest that livestock management can benefit from the knowledge from wildlife research gained in our study.

Statistical analyses of our data are, however, subject to the problems of pseudoreplication [63] as we could not find several exclosures of the same kind in our study area. Hence, we analyzed the data in view of [64] by ensuring both appropriate methods of analysis and limiting the result interpretation without generalizing them across other areas. Since empirical studies on the scale of our study are rare [65], our study provided a unique opportunity to gain insights across ecologically comparable sites into community and landscape aspects of plant-herbivore interactions and highlights important trends.

## Conclusions

Overall, our study showed that the selection of grass species by grazing herbivores was determined by grass nutrient content, palatability, and greenness. Further, herbivore composition and density had strong impacts on the species and structural heterogeneity of our study sites. Tall grass patches, often drastically reduced under high livestock grazing pressure, must be retained in grazing systems as dry season forage refuges and to promote landscape heterogeneity. Understanding these mechanisms of the interaction between herbivores and the herbaceous layer of the savannas helps inform management on decisions relating to the numbers and types of animals that can be supported by a specific livestock or wildlife grazing area.
